# High-Risk Human Papillomavirus Infection in Squamous Cell Carcinoma of the Larynx: A Study From a Tertiary Care Center in North India

**DOI:** 10.7759/cureus.34760

**Published:** 2023-02-08

**Authors:** Shreshtha Ghosh, Sanjeev Kumar, Rajiv Chaudhary, Priyadarshini Guha

**Affiliations:** 1 Pathology, Kalyan Singh Super Speciality Cancer Institute, Lucknow, IND; 2 Otolaryngology, Rani Durgavati Medical College (RDMC), Banda, IND; 3 Otolaryngology, Amaya ENT Clinic, Fatehpur, IND

**Keywords:** squamous cell carcinoma of the larynx, polymerase chain reaction (pcr), high-risk papillomavirus, head and neck oncology, smoking tobacco, pcr (polymerase chain reaction)

## Abstract

Background

Human papillomavirus (HPV) 16/18 infection has been considered as an important etiological factor for laryngeal carcinoma. Considering its impact on prognosis, it is important to understand the true prevalence of HPV-associated laryngeal squamous cell carcinoma (LSCC) in the northeast region of India.

Materials and methods

A prospective observational study was conducted among patients with laryngeal squamous cell carcinoma in the department of otolaryngology of King George's Medical University (KGMU), Lucknow.

Results

In this study, the total number of cases was 62. HPV 16/18 positivity was higher (p=0.02) among the age group 31-40 years (40%) as compared to above 50 years (3.3%). HPV positivity was higher among females (50%) as compared to males (12.5%). Out of 34 tobacco smokers, HPV positivity was seen in 20.6% as compared to no positivity among patients without any history of addiction.

HPV positivity was found in 19.6% of supraglottic cancer and no positivity among glottic cancer. HPV positivity was higher among the T1 stage of supraglottic carcinoma (40%) as compared to T3 (17.4%). In glottic cases, HPV positivity was nil in all T stages.

Conclusion

The association of tumor HPV status with laryngeal squamous cell carcinoma in females and young patients (<50 years) observed in our study is consistent with prior studies, and this reflects that HPV status should be considered in the design or analysis for the treatment of laryngeal cancer. We tried to highlight the importance of diagnosing HPV-positive laryngeal squamous cell carcinoma at early stages of the disease and also added information about the prevalence of HPV-positive LSCC in this zone of the country. We have observed that laryngeal carcinoma from tobacco smokers contains transcriptionally active HPV and hence may act as a risk factor or act synergistically with HPV infection. Further studies with larger sample size are needed to clearly establish the association of HPV in laryngeal squamous cell carcinoma and its impact on disease prognosis.

## Introduction

Squamous cell carcinoma of the head and neck is the most common cancer in Northeast India. Its incidence varies between 25% and 30% of all malignancies [1]. Common sites of involvement include the lip, tongue, buccal mucosa, nasopharynx, hypopharynx, and larynx.

The role of tobacco and alcohol is well established in the causation of head and neck squamous cell carcinoma in the Indian population. However, recent studies show human papillomavirus (HPV) as an emerging etiological agent in the pathogenesis of head and neck squamous cell carcinomas.

Although there is sufficient data establishing the role of HPV in head and neck squamous cell carcinoma in the western population, very few published studies exist for the Indian population. There is a paucity of literature on the role of HPV specifically in laryngeal squamous cell carcinoma (LSCC) in the Indian population as the majority of studies have included all the subsites of the head and neck. The study by Jacob et al. investigated the role of HPV exclusively in laryngeal squamous cell carcinoma, which demonstrated the prevalence of HPV in laryngeal cancer of about 34% [2].

Laryngeal cancer is the ninth and seventh most common cause of cancer in males in Asia and India, respectively. In India, the incidence of laryngeal cancer has been reported to be 1.26-8.18 per 100,000 population, in different regions in the country [3].

HPV 16/18 infection has been considered an important etiological factor in head and neck cancer [4,5]. However, unlike cervical carcinoma and oropharyngeal squamous cell carcinoma, the role of HPV 16/18 in the pathogenesis of laryngeal carcinoma has not been clearly defined [6,7]. The prevalence of HPV in laryngeal cancer shows wide variation within different zones of the same country [8,9]. Possible causes proposed are variations in demographic factors, tumor site, and method of virus detection.

Human papillomavirus (HPV) is a member of the papillomavirus family of viruses. High-risk HPV (16/18) is associated with laryngeal squamous cell carcinoma, other types of carcinomas are rare in the larynx, and no association with high-risk HPV (16/18) has been studied yet.

High-risk HPV types 16 and 18 produce oncogenic effect by the expression of E6 and E7 oncoprotein leading to the inhibition of p53, retinoblastoma protein (RB), and p16 tumor suppressor genes [10]. Cigarette smoking and alcohol are other risk factors that act synergistically with HPV and alter natural immune response, increasing the susceptibility to infections [11,12].

Demographic factors also play a pivotal role in the complex interaction of various genetic and environmental factors and may be associated with high incidence of high-risk HPV-positive laryngeal squamous cell carcinoma. The exact status of high-risk HPV in laryngeal squamous cell carcinoma is largely unknown in Northeast India. As HPV positivity is an important prognostic parameter, it is worthwhile to understand the true prevalence of HPV-associated laryngeal squamous cell carcinoma in this region of India.

## Materials and methods

Aims and objectives

We conducted this study with the goal of finding the prevalence of high-risk HPV infection among laryngeal squamous cell carcinoma patients and assessing the association of high-risk HPV with various demographic factors and the staging of laryngeal squamous cell carcinoma patients.

A prospective study was conducted among patients with laryngeal cancers who visited the outpatient department of otolaryngology, King George's Medical University (KGMU), Lucknow, India, for a duration of two years. The study was approved by King George's Medical University Ethics Committee, Lucknow, India, with reference number 32 ECM II A/P16. Informed consent was taken from all patients. A total of 62 cases of biopsy-proven laryngeal squamous cell carcinoma were included in this study. Cases with benign tumors and inadequate biopsy and patients with less than 18 years of age were excluded.

Based on the finding of the study done by Jacob et al., the pooled estimate of high-risk HPV infection among laryngeal squamous cell carcinoma considered was 34% [2]. Using this, with a 95% confidence interval (CI) and a margin of error of 12%, we found the sample size to be 60. But, in the total duration of our study, we got 62 cases that have been included in the present study.

A brief demographic history of addiction to alcohol, tobacco chewing, and tobacco smoking was taken. After laryngoscopic biopsy, the tissue was transferred in 10% neutral buffered formalin for histopathological diagnosis. A part of the tissue was stored at -80 degrees for HPV studies. HPV detection was done by an in-house real-time polymerase chain reaction (PCR) (application binary interface {ABI} system).

A detailed history of smoking, tobacco chewing, and alcohol consumption was taken. A defined smoking index (cigarettes/day×365 days) was calculated, and patients with a smoking index of 730 or more were considered smokers. Likewise, the frequency of tobacco chewed per day over the entire time period in years (chewing year) was calculated, and the patient with a chewing year of 365 or more was considered a tobacco chewer. Similarly, a history of regular alcohol consumption over the entire time period was calculated. Patients with a regular alcohol intake of 90 L in one year were considered an alcoholic in this study.

HPV DNA extraction and purification

TRIzol reagent followed by ethanol precipitation technique was used as for genomic DNA extraction from fresh-frozen paraffin-embedded (FFPE) tissues. In brief, interphase and organic phase were obtained after separating them from the upper aqueous phase. Three hundred microliter of 100% ethanol per 1 ml of TRIzol was mixed with interphase and organic phase, and after gentle vortexing, it was incubated at 15°C-30°C for 2-3 minutes. After incubation, the mixture was centrifuged for five minutes at 12,000 revolutions per minute (rpm) at temperature of 25°C±2°C. The pellet formed was washed twice in 0.1 M sodium citrate solution for 30 minutes at 15°C-30°C. A dry pellet obtained after was resuspended in 1 ml 75% ethanol/ml of TRIzol for 10-20 minutes at 15°C-30°C followed by centrifugation (12,000 rpm/minute). Dissolved DNA in Tris EDTA (TE) buffer was purified and quantified by Wizard Genomic DNA Purification Kit (Promega Corporation, Madison, WI) and Picodrop Spectrophotometer (Thermo Fisher Scientific, Waltham, MA) at 260/280 nm wavelength, respectively.

HPV 16/18 detection by real-time PCR

Real-time PCR (StepOne Real-Time PCR, Applied Biosystems, Waltham, MA) was carried out for the detection of HPV 16/18. MY09 and MY011 HPV consensus primers were used. For PCR reaction, a mixture of 20 μl was prepared by mixing 10 μl of 2X SYBR Green (deoxyribose nucleotide triphosphates {DNTPs}, ROX dye, and Taq polymerase), 1 μl of 10 μM of MY09/01, 1 μl of DNA, and dH_2_O. For DNA extraction, samples were loaded into a 48-well plate (Applied Biosystems) followed by a thermal cycle of 95°C for five minutes; 40 cycles of 95°C for 30 seconds, 56°C for 30 seconds, 72°C for 45 seconds, and finally 72°C for five minutes were carried out. The amplification of an 180 base pair (bp) fragment of the housekeeping gene β-actin was used for the validation of the quality of tissue DNA.

Statistical analysis

The results were presented in percentage. The chi-square test was used to assess the significance between HPV+/- by different variables. The relative risk with its 95% confidence interval (CI) was calculated to find out the risk of HPV positivity among variables. P<0.05 was considered as significant.

## Results

In this study, the total number of cases was 62.

Distribution of cases based on demographic factors

HPV positivity was significantly higher (p=0.02) among the cases of the age group 31-40 years (40%) as compared to 41-50 (26.9%) and above 50 years (3.3%) (Table 1).

**Table 1:** Demographic particulars of the sample The mean age of the cases was 52.34±4.67 years with a male preponderance (90.32%)

Demographic particulars	Frequency	Percentage
Age group		
21-30	1	1.61
31-40	5	8.06
41-50	26	41.94
>50	30	48.39
Gender		
Male	56	90.32
Female	6	9.68
Religion		
Hindu	48	77.42
Muslim	13	20.97
Others	1	1.61
Literacy status		
Literate	20	32.26
Illiterate	42	67.74
Addiction history		
Tobacco chewing	4	6.45
Tobacco smoking	34	54.84
Multiple addiction	23	37.09
Alcohol	0	0
No addiction	1	1.61

In this study, out of the total six female laryngeal squamous cell carcinoma cases, three (50%) were HPV 16/18-positive; however, among the 56 positive laryngeal squamous cell carcinoma cases, seven (12.5%) were HPV 16/18-positive. Hence, the HPV 16/18-positive rate among females was significantly higher when compared to males (Table 2). Out of the total 48 Hindu patients, eight were HPV-positive, and among the 13 Muslim patients, two were HPV-positive (Table 2).

**Table 2:** Demographic factors and HPV positivity HPV, human papillomavirus; PCR, polymerase chain reaction

Demographic particulars	HPV PCR			
	Positive (n=10)		Negative (n=52)	
	Number	Percentage	Number	Percentage
Age group				
21-30	0	0.0	1	100
31-40	2	40	3	60
41-50	7	26.9	19	73.1
>50	1	3.3	29	96.7
Gender				
Male	7	12.5	49	87.5
Female	3	50	3	50
Religion				
Hindu	8	16.7	40	83.3
Muslim	2	15.2	11	84.6
Others	0	0	1	100
Literacy status				
Literate	5	25	15	75
Illiterate	5	11.9	37	88.1

Out of the 34 tobacco smoker patients, HPV positivity was seen in 20.6% of patients as compared to patients exposed to multiple risk factors (13%). There was no positivity among patients without any history of addiction (Table 3).

**Table 3:** Addiction history and HPV positivity HPV, human papillomavirus; PCR, polymerase chain reaction

Demographic particular	HPV PCR				
	Positive (n=10)			Negative (n=52)	
	Number	Percentage	Number		Percentage
Addiction history					
Tobacco chewing	0	0	4		100
Tobacco smoking	7	20.6	27		79.4
Alcohol	0	0	0		0
Multiple	3	13.0	20		87
No addiction	0	0	1		100

The distribution of cases based on the subsite of laryngeal cancer and their association with high-risk HPV

A total of 51 (82.2%) patients had supraglottic laryngeal cancer, and 11 (17.8%) patients had glottic laryngeal cancer. High-risk HPV positivity was found in 19.6% of the supraglottic cancer and no positivity among glottic cancer (Table 4).

**Table 4:** HPV result based on the subsite of the larynx HPV positivity was found in 19.6% of the supraglottic patients, and no positivity was seen among the glottic HPV: human papillomavirus

Region	HPV+ (%)	HPV- (%)	Total
Supraglottis	10 (19.6)	41 (80.4)	51
Glottis	0 (0)	11 (100)	11

The distribution of high-risk HPV-positive cases based on T staging in different subsites of laryngeal carcinoma

On classifying supraglottic tumor by tumor-node-metastasis (TNM), HPV positivity was insignificantly higher (p>0.05) among the T1 stage of supraglottic carcinoma (40%) as compared to T4 (36.4%), T3 (17.4%), and T2 (0.0%) (Table 5). Out of the 11 glottic cases, HPV positivity was nil in all T stages (Table 5).

**Table 5:** HPV positivity by the T stage of supraglottic and glottic carcinoma HPV positivity was higher among the T1 stage of supraglottic carcinoma (40%) as compared to T4 (36.4%), T3 (17.4%), and T2 (0.0%). HPV positivity was nil among the T stage of glottic carcinoma (40%) patients HPV: human papillomavirus

	HPV+ (%)	HPV- (%)	Total
T stage (supraglottis)			
T1	2 (40)	3 (60)	5
T2	0 (0)	12 (100)	12
T3	4 (17.4)	19 (82.6)	23
T4	4 (36.4)	7 (63.6)	11
T stage (glottis)			
T1	0	0	0
T2	0	6 (100)	6
T3	0	3 (100)	3
T4	0	2 (100)	2

The distribution of high-risk HPV-positive cases based on N staging in different subsites of laryngeal carcinoma

Table 6 shows HPV positivity by the N stage of glottic and supraglottic carcinoma.

**Table 6:** HPV positivity by the N stage of supraglottic and glottic carcinoma HPV positivity was higher among the N3 stage of supraglottic carcinoma (33.3%) patients as compared to N2 (30%), N0 (14.8%), and N1 (12.5%). HPV positivity was nil among the N stage of glottic carcinoma (40%) patients HPV: human papillomavirus

	HPV+ (%)	HPV- (%)	Total
N stage (supraglottis)			
N0	4 (14.8)	23 (85.2)	27
N1	1 (12.5)	7 (87.5)	8
N2	3 (30)	7 (70)	10
N3	2 (33.3)	4 (66.7)	6
N stage (glottis)			
N0	0	7 (100)	7
N1	0	2 (100)	2
N2	0	1 (100)	1
N3	0	1 (100)	1

## Discussion

It has been found that high-risk HPV infection is biologically relevant in laryngeal carcinogenesis that manifested as increased expression of the p16 protein. A broad range of prevalence has been noted in individual studies; approximately 25% of laryngeal squamous cell carcinomas are associated with HPV infection [13]. Similar to oral squamous cell carcinoma, HPV 16 is the most prevalent genotype for HPV-positive laryngeal squamous cell carcinoma [8,14].

Laryngeal squamous cell carcinoma is a multifactorial disease. The consumption of alcohol and cigarette smoking are the predominant etiological factors. Carcinogens such as tar and nicotine present in cigarette smoke and alcohol alter the immune response and cause increased predisposition to acquire HPV infection [11,12]. Despite this knowledge, the clinical significance of these infections and risk factors and their implications on disease prevention and treatment are unclear. Also, there is a lack of specific predictive/prognostic marker for laryngeal squamous cell carcinoma [15]. These lacunas in the laryngeal squamous cell carcinoma still remain the subject of debate and hence require further investigation [16]. As compared to oropharyngeal cancer (82%), the association of HPV infection with laryngeal cancer is less prevalent (about 16%) [16]. Our results add further information to the previous HPV DNA studies of laryngeal squamous cell carcinoma. We were able to detect the presence of HPV DNA in 10 of the 62 cases (16%), which is similar to the study done by Brandwein et al. [17] and Hernandez et al. [18].

HPV positivity was higher among patients of <50 years of age. Younger patients are prone to more high-risk activities such as smoking and drinking, which could be one of the reasons for high HPV-positive rate. Further studies are required to prove the causal relation.

In this study, out of the total six female laryngeal squamous cell carcinoma cases, three (50%) were HPV 16/18-positive; however, among the 56 positive laryngeal squamous cell carcinoma cases, seven (12.5%) were HPV 16/18-positive. Similar findings were postulated by Ogura et al. [19].

In the overview, studies have reported that HPV-based cancers are significantly higher among males when compared to females [20]. These variations in findings need larger longitudinal studies for a possible explanation.

It was also observed that increased HPV positivity was noted among tobacco smoker patients (20.6%) as compared to patients exposed to multiple risk factors (13%) with nil positivity among nonaddicted patients. Similar to our study, Gomaa et al. showed that tobacco chewers, tobacco smokers, and alcoholics had significantly increased risk of HPV 16 infection [21]. In contrast to this, the studies done by Ralli et al. [22] and Kumar et al. [23] showed no statistically significant association between p16 expression and smoking or alcohol consumption.

In our study, HPV positivity was found in supraglottic carcinoma and no positivity in glottic carcinoma, in contrast to the results of previous studies [24], except for the study done by Morshed et al., which showed similar results [25].

HPV positivity was higher among the T1 stage of supraglottic carcinoma (40%) as compared to T4 (36.4%), T3 (17.4%), and T2 (0.0%), which was in harmony with the results of the study done by Jitani et al. [26]. However, Singh et al. [27] and Chaudhary et al. [28] demonstrated higher HPV positivity in T3/T4 stage compared to T1/T2 stage. In addition, HPV positivity was higher among the N3 stage of supraglottic carcinoma patients (33.3%) as compared to N2 (30%), N0 (14.8%), and N1 (12.5%).

There are conflicting reports in the literature on the association of HPV positivity with disease outcome. In a review by Nair et al. [29], they showed that HPV-positive head and neck cancers had better survival with improved prognosis; however, no data was available exclusively for laryngeal squamous cell carcinoma in their study. They suggested de-intensification of treatment for patients with HPV-positive oropharyngeal squamous carcinoma, as they are typically younger and have an improved survival rate, compared with other patients with the same disease. Whether or not the de-escalation of treatment can be achieved in laryngeal cancer patients should be studied as it would greatly enhance the quality of life.

The study done by Yang et al. in 2021 showed that HPV positivity and programmed death-ligand 1 (PD-L1) were associated with better prognosis [30]. Another study done by Peralta et al. showed that the presence of HPV in patients of epidermal growth factor receptor (EGFR)-positive laryngeal squamous cell carcinoma (LSCC) was associated with better prognosis than LSCC with EGFR overexpression only, thus highlighting its role in the etiopathogenesis of laryngeal squamous cell carcinoma [31]. The prognostic role for HPV in laryngeal squamous cell carcinoma was also as found in a study done by Wang et al. [32] where HPV positivity was observed in the early stage of LSCC. In the present study, no definite conclusion can be drawn regarding the prognostic significance of high-risk HPV in squamous cell carcinoma of the larynx due to the limited sample size. Larger longitudinal studies are required to prove the prognostic significance of HPV 16/18-positive LSCC. However, to date, treatment decision is not based on HPV status in squamous cell carcinoma of the larynx.

## Conclusions

The association of tumor HPV status with laryngeal SCC in female and young patients (<50 years) observed in our study is consistent with prior studies. We have tried to highlight the importance of diagnosing HPV-positive laryngeal carcinoma at the early stages of the disease. We have observed that laryngeal carcinoma from tobacco smokers contains transcriptionally active HPV and hence may act as a risk factor or act synergistically with HPV infection. However, more robust and extensive studies with large sample sizes are needed to clearly establish the association of HPV in laryngeal squamous cell carcinoma and its impact on disease prognosis.

## References

[1] Ferlay J, Colombet M, Soerjomataram I (2019). Estimating the global cancer incidence and mortality in 2018: GLOBOCAN sources and methods. Int J Cancer.

[2] Jacob SE, Sreevidya S, Chacko E, Pillai MR (2002). Cellular manifestations of human papillomavirus infection in laryngeal tissues. J Surg Oncol.

[3] Sung H, Ferlay J, Siegel RL, Laversanne M, Soerjomataram I, Jemal A, Bray F (2021). Global cancer statistics 2020: GLOBOCAN estimates of incidence and mortality worldwide for 36 cancers in 185 countries. CA Cancer J Clin.

[4] Sasidharanpillai S, Ravishankar N, Kamath V, Bhat PV, Bhatt P, Arunkumar G (2021). Prevalence of human papillomavirus (HPV) DNA among men with oropharyngeal and anogenital cancers: a systematic review and meta-analysis. Asian Pac J Cancer Prev.

[5] Allegra E, Bianco MR, Mignogna C, Caltabiano R, Grasso M, Puzzo L (2021). Role of p16 expression in the prognosis of patients with laryngeal cancer: a single retrospective analysis. Cancer Control.

[6] Wierzbicka M, Klussmann JP, San Giorgi MR, Wuerdemann N, Dikkers FG (2021). Oral and laryngeal HPV infection: incidence, prevalence and risk factors, with special regard to concurrent infection in head, neck and genitals. Vaccine.

[7] Jun HW, Ji YB, Song CM, Myung JK, Park HJ, Tae K (2021). Positive rate of human papillomavirus and its trend in head and neck cancer in South Korea. Front Surg.

[8] Liu B, Lu Z, Wang P, Basang Z, Rao X (2010). Prevalence of high-risk human papillomavirus types (HPV-16, HPV-18) and their physical status in primary laryngeal squamous cell carcinoma. Neoplasma.

[9] Al-Qudah MA, Al-Shaikh AF, Haddad HK (2020). Prevalence and detection of sexually transmitted cases of laryngeal carcinoma. Head Neck Pathol.

[10] Jiang S, Dong Y (2017). Human papillomavirus and oral squamous cell carcinoma: a review of HPV-positive oral squamous cell carcinoma and possible strategies for future. Curr Probl Cancer.

[11] Halec G, Holzinger D, Schmitt M (2013). Biological evidence for a causal role of HPV16 in a small fraction of laryngeal squamous cell carcinoma. Br J Cancer.

[12] Mehta H, Nazzal K, Sadikot RT (2008). Cigarette smoking and innate immunity. Inflamm Res.

[13] Nandi S, Mandal A, Chhebbi M (2021). The prevalence and clinicopathological correlation of human papillomavirus in head and neck squamous cell carcinoma in India: a systematic review article. Cancer Treat Res Commun.

[14] Kreimer AR, Bhatia RK, Messeguer AL, González P, Herrero R, Giuliano AR (2010). Oral human papillomavirus in healthy individuals: a systematic review of the literature. Sex Transm Dis.

[15] Torrente MC, Rodrigo JP, Haigentz M Jr (2011). Human papillomavirus infections in laryngeal cancer. Head Neck.

[16] Sánchez Barrueco A, González Galán F, Villacampa Aubá JM (2019). P16 influence on laryngeal squamous cell carcinoma relapse and survival. Otolaryngol Head Neck Surg.

[17] Brandwein MS, Nuovo GJ, Biller H (1993). Analysis of prevalence of human papillomavirus in laryngeal carcinomas. Study of 40 cases using polymerase chain reaction and consensus primers. Ann Otol Rhinol Laryngol.

[18] Hernandez BY, Goodman MT, Lynch CF (2014). Human papillomavirus prevalence in invasive laryngeal cancer in the United States. PLoS One.

[19] Ogura H, Watanabe S, Fukushima K, Masuda Y, Fujiwara T, Yabe Y (1991). Presence of human papillomavirus type 18 DNA in a pharyngeal and a laryngeal carcinoma. Jpn J Cancer Res.

[20] Näsman A, Du J, Dalianis T (2020). A global epidemic increase of an HPV-induced tonsil and tongue base cancer - potential benefit from a pan-gender use of HPV vaccine. J Intern Med.

[21] Gomaa MA, El Gindy KE, Nabi UG, Mohammed HM, Twab NA, Mahmoud R, Mohiy KM (2017). Human papillomavirus subtype 16 and the pathologic characteristics of laryngeal cancer. OTO Open.

[22] Ralli M, Singh S, Yadav SP, Sharma N, Verma R, Sen R (2016). Assessment and clinicopathological correlation of p16 expression in head and neck squamous cell carcinoma. J Cancer Res Ther.

[23] Kumar R, Rai AK, Das D (2015). Alcohol and tobacco increases risk of high risk HPV infection in head and neck cancer patients: study from north-east region of India. PLoS One.

[24] Hoshikawa T, Nakajima T, Uhara H (1990). Detection of human papillomavirus DNA in laryngeal squamous cell carcinomas by polymerase chain reaction. Laryngoscope.

[25] Morshed K, Korobowicz E, Szymański M, Skomra D, Gołabek W (2005). Immunohistochemical demonstration of multiple HPV types in laryngeal squamous cell carcinoma. Eur Arch Otorhinolaryngol.

[26] Jitani AK, Raphael V, Mishra J, Shunyu NB, Khonglah Y, Medhi J (2015). Analysis of human papilloma virus 16/18 DNA and its correlation with p16 expression in oral cavity squamous cell carcinoma in north-eastern India: a chromogenic in-situ hybridization based study. J Clin Diagn Res.

[27] Singh V, Husain N, Akhtar N (2015). Do human papilloma viruses play any role in oral squamous cell carcinoma in north Indians?. Asian Pac J Cancer Prev.

[28] Chaudhary AK, Pandya S, Mehrotra R, Bharti AC, Singh M, Singh M (2010). Comparative study between the Hybrid Capture II test and PCR based assay for the detection of human papillomavirus DNA in oral submucous fibrosis and oral squamous cell carcinoma. Virol J.

[29] Nair D, Mair M, Singh A, D’Cruz A (2018). Prevalence and impact of human papillomavirus on head and neck cancers: review of Indian studies. Indian J Surg Oncol.

[30] Yang SM, Wu M, Han FY, Sun YM, Yang JQ, Liu HX (2021). Role of HPV status and PD-L1 expression in prognosis of laryngeal squamous cell carcinoma. Int J Clin Exp Pathol.

[31] Peralta R, Garcia P, Valdivia A (2018). HPV could be a potential factor of survival in laryngeal cancer: a preliminary study in Mexican patients. Asian Pac J Cancer Prev.

[32] Wang H, Zhang Z, Sun R, Lin H, Gong L, Fang M, Hu WH (2015). HPV infection and anemia status stratify the survival of early T2 laryngeal squamous cell carcinoma. J Voice.

